# Unexpected Lung and Brain Metastases 9 Years After Thyroid Lobectomy for Follicular Adenoma: A Case Report

**DOI:** 10.3389/fendo.2019.00783

**Published:** 2019-11-12

**Authors:** Yoo Jin Lee, Dong Wook Kim, Gi Won Shin, Young Jin Heo, Jin Young Park, Jin Wook Baek, Hye Jung Choo, Young Jun Cho, Ha Kyoung Park, Tae Kwun Ha, Do Hun Kim, Soo Jin Jung, Ji Sun Park, Sung Ho Moon, Ki Jung Ahn

**Affiliations:** ^1^Department of Radiology, Busan Paik Hospital, Inje University College of Medicine, Busan, South Korea; ^2^Department of General Surgery, Busan Paik Hospital, Inje University College of Medicine, Busan, South Korea; ^3^Department of Otorhinolaryngology-Head and Neck Surgery, Busan Paik Hospital, Inje University College of Medicine, Busan, South Korea; ^4^Department of Pathology, Busan Paik Hospital, Inje University College of Medicine, Busan, South Korea; ^5^Department of Nuclear Medicine, Busan Paik Hospital, Inje University College of Medicine, Busan, South Korea; ^6^Department of Anesthesiology and Pain Medicine, Busan Paik Hospital, Inje University College of Medicine, Busan, South Korea; ^7^Department of Radiation Oncology, Busan Paik Hospital, Inje University College of Medicine, Busan, South Korea

**Keywords:** thyroid, papillary thyroid carcinoma, follicular adenoma, metastasis, ultrasonography

## Abstract

**Background:** Benign thyroid follicular tumors without histological evidence of carcinoma can metastasize. However, the pathogenesis of metastasis remains unclear. Here, the new proposed terminology, “non-invasive follicular thyroid neoplasm with papillary-like nuclear features” should be considered. We present a case of an encapsulated type of follicular variant of papillary thyroid carcinoma (FVPTC) that exhibited distant lung and brain metastases and was initially diagnosed as follicular adenoma.

**Case Report:** In December 2006, a 64-year-old woman underwent ultrasonography-guided fine-needle aspiration of the right thyroid nodule at our hospital because of a palpable right neck mass. Right lobectomy was performed, and a follicular adenoma was diagnosed. In October 2015, she visited our hospital owing to dry cough and mild dyspnea and underwent computed tomography-guided transthoracic core needle biopsy for the lung nodule owing to probably multiple lung metastasis on chest X-ray and computed tomography. Based on retrospective analysis of the primary thyroid tumor and lung nodule specimen, an encapsulated follicular variant of papillary thyroid carcinoma with lung metastasis was confirmed.

**Conclusion:** We report a case of an encapsulated follicular variant of papillary thyroid carcinoma with unexpected metastasis to the lung 9 years after thyroid surgery in a patient who was initially diagnosed as follicular adenoma. A careful close follow-up with re-examination of the histopathology specimen may be needed in patients who were diagnosed with benign thyroid follicular tumors.

## Introduction

According to several studies, encapsulated thyroid follicular tumors without histological evidence of carcinoma can metastasize (1–5); these are referred to as metastasizing adenomas (2). The types of benign lesions that may undergo distant metastasis include benign nodular goiter, follicular adenoma, and benign oncocytic follicular thyroid tumor (1–5). In a previous study, 5 (0.17%) of 2,975 adenomatous thyroid nodules without pathological evidence of carcinoma exhibited metastases to lymph nodes or distant organs (1). Another report discussed a case of brain metastasis from a thyroid adenomatous nodule or an encapsulated thyroid follicular tumor without capsular and vascular invasion (3). In a recent case report, investigators reported a case of late bone metastasis from an apparently benign oncocytic follicular thyroid tumor (5). These researchers suggested that benign thyroid nodule or encapsulated follicular tumor without capsular and vascular invasion can metastasize (1, 3–5). However, there is no evidence regarding the pathogenesis of distant metastasis from these benign thyroid lesions. To focus on this issue, we present a case of an encapsulated type of follicular variant of PTC (FVPTC) that exhibited distant lung and brain metastases and was initially diagnosed as follicular adenoma.

## Case Reports

In December 2006, a 64-year-old woman with a palpable mass on the right side of her neck visited a local clinic and was referred to our hospital. She had no past medical history or risk factors for thyroid malignancy. Results of the thyroid function tests were as follows: T3, 164.7 ng/dL (normal range, 80–200 ng/dL); free T4, 1.28 ng/dL (normal range, 0.93–1.71 ng/dL); and thyroid-stimulating hormone, 1.02 mIU/L (normal range, 0.27–4.20 mIU/L).

**Figure 1:**
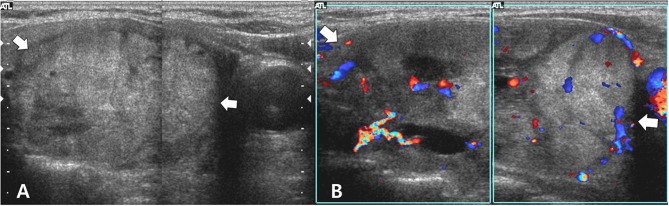
Longitudinal gray-scale **(A)** and color Doppler **(B)** sonograms show a predominantly solid thyroid nodule (arrows, 5.5 cm in the largest diameter) in the right lobe. This nodule exhibits benign sonographic features such as isoechogenicity, smooth margins, a hypoechoic halo, oval shape, and peripheral vascularity. Based on histopathological analysis after the right lobectomy, the right thyroid nodule was diagnosed as follicular adenoma.

To evaluate thyroid nodules, thyroid ultrasonography (US) was performed by a radiologist with 4 years of experience using a high-resolution ultrasound instrument (HDI 5000; Philips Medical System, Bothell, WA, USA) equipped with a 5–12 MHz linear probe. Thyroid US showed a predominantly solid thyroid nodule with benign US features in the right lobe (Figures 1A,B). This nodule exhibited no malignant US features such as hypoechogenicity, spiculated/microlobulated margins, microcalcification, and non-parallel orientation (taller-than-wide). In the left lobe, two solid thyroid nodules with similar US features were found. For further evaluation, US-guided fine-needle aspiration was performed for the largest thyroid nodule in the right lobe. Cytology revealed “atypia of undetermined significance” (Bethesda category III). For the diagnosis and treatment of this lesion, a right lobectomy and a left nodulectomy were performed. For the histopathological analysis, the tumor was diagnosed as a follicular adenoma in the right lobe. In addition, two areas of nodular hyperplasia were found in the left lobe. The patient did not follow-up for 9 years after surgery, because of follow-up loss. During this period, the patient took thyroxine replacement therapy in the local clinic.

**Figure 2:**
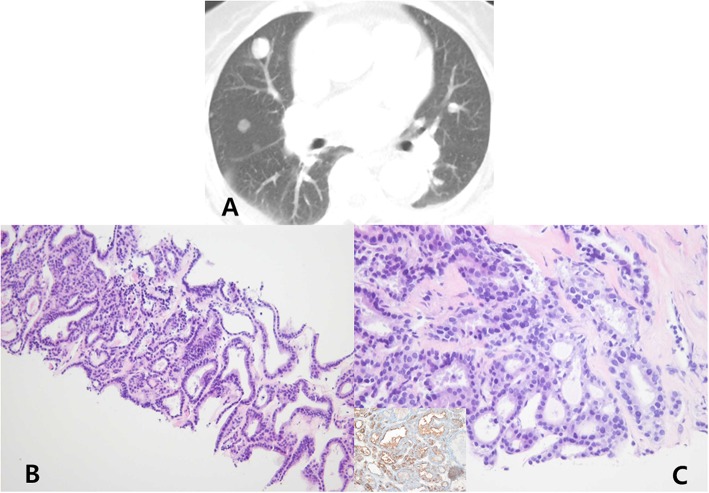
Chest computed tomography (lung window) **(A)** show multiple, variable-sized nodules in both the lungs. Computed tomography-guided transthoracic core needle biopsy was performed for the lung nodule in the right upper lobe. The biopsied lung mass was composed of cells with an irregular follicular pattern (hematoxylin and eosin stain, ×100) **(B)**. The follicular cells show nuclear enlargement, irregularity, and grooves (hematoxylin and eosin stain, ×400), which were positive for thyroglobulin, thyroid transcription factor-1, and HBME-1 immunohistochemical stain (inset) **(C)**.

In October 2015, she revisited our hospital with dry cough and mild dyspnea. A subsequent chest X-ray and computed tomography (CT) showed multiple lung nodules in both lungs (Figure 2A). For further evaluation, a neck US was performed, but no mass or lymphadenopathy was observed in the remnant left thyroid and other neck area. A CT-guided transthoracic core needle biopsy was performed for the lung nodule in the right upper lobe. For the biopsied tissue, hematoxylin and eosin staining and immunohistochemical staining for the biopsied tissue were performed. The biopsied lung mass revealed follicular patterned tumor cells which were positive for thyroglobulin, thyroid transcription factor-1, and HBME-1 (Figures 2B,C), and histopathological analysis confirmed lung metastasis from thyroid malignancy. In this time, the serum thyroglobulin level was > 500 ng/mL.

**Figure 3:**
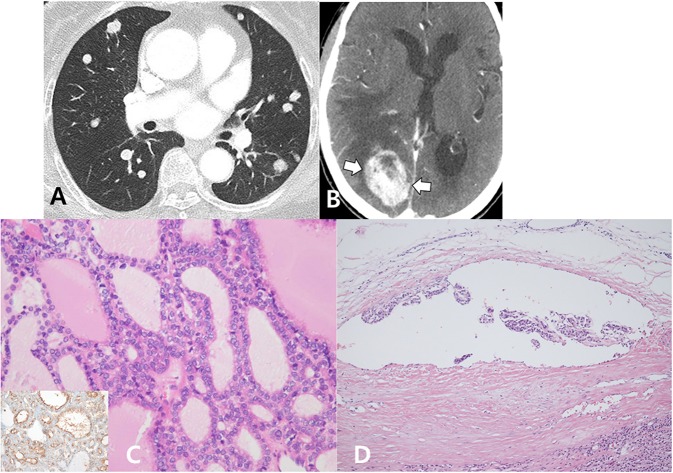
Multiple metastatic nodules in both the lungs exhibited interval increase in size and number on follow-up chest CT **(A)**. On the same day, the patient underwent neck CT, and a newly developed heterogeneous enhancing mass (arrows) was detected in the right parietal lobe **(B)**. In the re-evaluation of a previous right lobectomy specimen, the right thyroid mass was entirely composed of small- and large-sized follicles and diffusely presented with papillary-like nuclear features (hematoxylin and eosin stain, ×400), which were positive for HBME-1 immunohistochemical staining (inset) **(C)**. A focus of discrete vascular invasion with endothelium covered tumor cells adherent to the vessel wall (hematoxylin and eosin stain, ×200) is identified **(D)**.

In November 2015, a non-enhanced brain CT was performed following the patient's request, and no abnormal lesions were found in the brain. In March 2018, follow-up chest X-ray and CT were performed (Figure 3A), and multiple metastatic lung nodules exhibited interval increase in size and number. On the same day, the patient underwent neck CT to rule out primary malignancies. Although a right parietal lobe mass was incidentally identified in the neck CT (Figure 3B), a biopsy trial for the right parietal lobe mass was not performed.

In April 2018, serial and deeper paraffin block sections of the previous thyroid tumor were prepared, and a pathologist reviewed the slides. The right thyroid mass was encapsulated and entirely composed of small and large sized follicles. The follicular cells revealed papillary-like nuclear features and positive for HBME-1 immunohistochemical stain. A focus of vascular invasion with endothelium covered tumor cells adherent to the vessel wall was also identified on serial section of thyroid tumor (Figures 3C,D). Finally, the diagnosis of the right thyroid mass was revised as encapsulated FVPTC, and this resulted in lung and brain metastases. Thus, we planned a treatment that included external radiation and radioactive iodine ablation after completing thyroidectomy. However, the patient refused these treatments because she could not accept the change in the diagnostic results. At present, no patient follow-up is available.

## Discussion

In the literature, many thyroid nodules previously diagnosed as follicular adenoma have been labeled as non-invasive encapsulated FVPTC owing to the lack of agreement on the minimal nuclear alteration criteria required to diagnose PTC (6, 7). It is likely that areas of microinvasion were not included in the examined histological sections because of limitations in histopathological tumor sampling (3–5). In addition, the new proposed terminology, “non-invasive follicular thyroid neoplasm with papillary-like nuclear features” (NIFTP), which has key histopathologic features [i.e., lack of invasion, follicular growth pattern, and nuclear features of papillary thyroid carcinoma (PTC)] should be considered (6). Furthermore, considerable interobserver variability may be involved in the diagnosis of FVPTC (7). According to a recent study, the diagnosis of NIFTP/encapsulated FVPTC with invasion should be made based on careful and extensive review of the tumor capsule interface to exclude minimal invasion, such as the approach previously used to diagnose encapsulated follicular adenoma/follicular carcinoma (6).

In our case, FVPTC exhibited no malignant US findings. Histopathological examination revealed that the tumor was encapsulated by a thick fibrous capsule, and this might have corresponded to the smooth margin with a hypoechoic halo on US. However, US examinations can be limited when assessing follicular adenomas, follicular thyroid carcinoma, and FVPTC because these tumors tend to exhibit no malignant US findings, unlike classic PTC (8). In particular, follicular thyroid adenoma and carcinoma could be differentiated only on the basis of histopathology (6).

## Concluding Remarks

We experienced a case of encapsulated FVPTC with unexpected metastasis to the lung and brain 9 years after thyroid surgery to remove a mass that was initially diagnosed as follicular adenoma after surgery. Based on our findings, additional histopathological analysis with careful observation of cases initially diagnosed as benign thyroid tumors may be necessary. In addition, awareness regarding imaging features and histopathological findings of NIFTP may be necessary to appropriately manage NIFTP.

## Data Availability Statement

The raw data supporting the conclusions of this manuscript will be made available by the authors, without undue reservation, to any qualified researcher.

## Ethics Statement

This study was approved by the institutional review board of Busan Paik Hospital (IRB-18-0137), which waived the need for informed patient consent because of patient follow-up loss and the retrospective investigation involving the use of anonymized patient data. All procedures performed in this study involving human participants were in accordance with the ethical standards of the institutional and/or national research committee and with the 1964 Helsinki declaration and its later amendments or comparable ethical standards.

## Author Contributions

YL and DK wrote the manuscript. All authors listed have made a substantial, direct and intellectual contribution to the work, and approved it for publication.

### Conflict of Interest

The authors declare that the research was conducted in the absence of any commercial or financial relationships that could be construed as a potential conflict of interest.
